# Single-Trial MEG Data Can Be Denoised Through Cross-Subject Predictive Modeling

**DOI:** 10.3389/fncom.2021.737324

**Published:** 2021-11-11

**Authors:** Srinivas Ravishankar, Mariya Toneva, Leila Wehbe

**Affiliations:** ^1^IBM-Research, Yorktown Heights, NY, United States; ^2^Machine Learning Department, Carnegie Mellon University, Pittsburgh, PA, United States; ^3^Neuroscience Institute, Carnegie Mellon University, Pittsburgh, PA, United States

**Keywords:** MEG, single-trial, denoising, predictive modeling, shared response, N400m, naturalistic

## Abstract

A pervasive challenge in brain imaging is the presence of noise that hinders investigation of underlying neural processes, with Magnetoencephalography (MEG) in particular having very low Signal-to-Noise Ratio (SNR). The established strategy to increase MEG's SNR involves averaging multiple repetitions of data corresponding to the same stimulus. However, repetition of stimulus can be undesirable, because underlying neural activity has been shown to change across trials, and repeating stimuli limits the breadth of the stimulus space experienced by subjects. In particular, the rising popularity of naturalistic studies with a single viewing of a movie or story necessitates the discovery of new approaches to increase SNR. We introduce a simple framework to reduce noise in single-trial MEG data by leveraging correlations in neural responses across subjects as they experience the same stimulus. We demonstrate its use in a naturalistic reading comprehension task with 8 subjects, with MEG data collected while they read the same story a single time. We find that our procedure results in data with reduced noise and allows for better discovery of neural phenomena. As proof-of-concept, we show that the N400m's correlation with word surprisal, an established finding in literature, is far more clearly observed in the denoised data than the original data. The denoised data also shows higher decoding and encoding accuracy than the original data, indicating that the neural signals associated with reading are either preserved or enhanced after the denoising procedure.

## 1. Introduction

Naturalistic stimuli are becoming increasingly more common in cognitive neuroscience (Nishimoto et al., 2011; Wehbe et al., 2014a; Huth et al., 2016; Sonkusare et al., 2019; Hamilton and Huth, 2020; Nastase et al., 2020). These stimuli are often presented only once to each participant in order to maximize the diversity of the data recorded in a fixed session, thereby sampling the stimulus space broadly (Nishimoto and Gallant, 2011; Nishimoto et al., 2011) and limiting the effect of habituation and repetition suppression. However, a common challenge for single-trial brain recordings is the magnitude of noise present in the data (Blankertz et al., 2011). Classical brain imaging studies (Coles and Rugg, 1995; Hansen et al., 2010) rely on increasing the signal-to-noise ratio (SNR) by averaging the brain recordings over multiple trials of the same stimulus. The paradigm shift toward naturalistic stimuli requires new ways of analyzing the resulting brain recordings.

In this work, we propose a framework to alleviate the presence of noise in single-trial brain recordings without the need for multiple trials. We achieve this denoising by aggregating data from multiple participants who experience the same set of naturalistic stimuli. We demonstrate the use of this framework for denoising Magnetoencephalography (MEG) data from a naturalistic reading comprehension task. While MEG's high temporal resolution makes it attractive for studying the dynamics of processing of naturalistic stimuli, its SNR is low (Vrba and Robinson, 2001).

As mentioned above, the established strategy for reducing noise in MEG is to average across multiple trials of each stimulus, but this poses several problems: (1) Across multiple tasks, stimulus repetition has been shown to lead to priming (Henson, 2003). Priming refers to a behavioral change associated with an improvement in task-related performance when stimuli are repeated. In many cases, this is correlated with a reduction in the neural activity, called Repetition Suppression (Grill-Spector et al., 2006). More generally, the underlying activity across trials changes. (2) Naturalistic studies generally involve stimuli that take a significant amount of time to experience, such as a story or a movie. Requiring multiple repetitions of these stimuli limits the breadth of the overall stimulus space that can be sampled.

These challenges motivate the need for methods that can denoise MEG data with few repetitions or even single trials. We consider a setting where MEG data is acquired from multiple subjects as they read the same natural language story, with the same timing per word, and where the story is only shown once to the subjects, effectively making the experiment a single trial experiment. With multiple subjects processing the same stimulus, we hypothesize that the fraction of a subject's MEG signal that can be reliably modeled from other subjects is driven by the stimulus, and not noise. We propose a framework that leverages these cross-subject correspondences to denoise MEG data. Concretely, we predict each subject's data from every other subject, and aggregate the different estimates of the target subject to produce a denoised version of the target's data. We term this approach pairwise mapping (PM).

Our framework is close to hyperalignment (Haxby et al., 2011) and the Shared Response Model (SRM) (Chen et al., 2015) in that all approaches aggregate data from multiple subjects. However, these methods have been employed primarily in studies involving fMRI data (Xu et al., 2012; Baldassano et al., 2017; Yousefnezhad et al., 2020), and do not explicitly seek to denoise the data. MEG has much lower SNR, and to the best of our knowledge, our work is the first to show that single-trial naturalistic MEG data can be denoised successfully.

In hyperalignment, each subject is transformed from its anatomical space to a shared ‘information' space, under the constraint of preserving geometry between points in each space. The dimensionality of this shared space can be either equal to the original dimensions of the data (or a reference brain) such as in Guntupalli et al. (2016, 2018), Haxby et al. (2011), or can be a low-dimensional space to reduce noise or overfitting. For the latter case, however, Chen et al. (2015) showed that the mathematical formulation proposed in Haxby et al. (2011) may lead to a non-generalizable/uninformative solution, and proposed an alternative formulation that behaves well when the shared response lives in a low-dimensional space. This formulation and the parameters estimated as a result are termed the Shared Response Model (SRM).

Chen et al. (2015) refers to the SRM as a refinement of hyperalignment, formulated as a probabilistic framework. More concretely, they consider 2 mathematical formulations to optimize, with the first formulation originally proposed by Haxby et al. (2011). This distinction appears due to the choice of optimizing the objective either in individual subject space or in the shared information space, i.e., projecting from subject space to shared space, or vice versa. They show that when the shared space is of reduced dimensionality, their alternative formulation leads to empirically better solutions. This reduced dimensionality of the shared response is also noted to have a denoising effect, leading us to empirically compare the SRM's ability to denoise data with the PM approach that we propose. We find that the PM approach is better able to model the original MEG data than the SRM approach.

Alignment of data across subjects is also an active area of research in Transfer Learning (TL) literature in BCI (Wu et al., 2020). However, the end goal of these TL approaches differs from the goal of our work. While TL approaches attempt to improve the performance of a classifier or regression model in a target domain by utilizing examples in a source domain, our goal is more general than classification/regression: We seek to obtain a new version of the data that amplifies stimulus-related effects by minimizing stimulus-unrelated noise. This would enable neuroscientists to study these stimulus-related effects more easily. Our framework generates a version of the experimental data on which many different analyses can be carried out, not limited to decoding.

Of the TL approaches, Euclidean-space alignment (EA) (He and Wu, 2019) is closest to our goal because it aligns the data in Euclidean space, after which further analysis can be performed. However, EA and related approaches such as CORAL (Sun et al., 2017) are designed for unsupervised TL. Our setting has a significant number of labeled examples in both source *and* target domains; which is fairly atypical in TL literature. Thus approaches like EA or CORAL attempt to align second-order characteristics of the two domains, such as covariance matrices of the two respective feature spaces. On the other hand, we directly learn a mapping between the feature spaces in a supervised regression, instead of relying on such second-order characteristics. In this sense, our setting and approach shares some similarity to Style Transfer Mapping (STM), a component of the supervised TL approach introduced in Li et al. (2019). However, rather than mapping the data directly between feature spaces, they choose to map some derived representations that are directly applicable to classifiers in the target domain. On the other hand, we map the raw MEG data between subjects in order to enhance the task-relevant signal in it, and carry out further analysis. In the interest of rigor, we include some experiments and comparison of our method with EA-aligned data in the Appendix.

## 2. Methods

### 2.1. MEG Data

We use the dataset from Wehbe et al. (2014b) and Toneva et al. (2020). It consists of MEG data collected from 8 native English speakers (4 females and 4 males) aged 18–40 years, who gave their written informed consent approved by the Carnegie Mellon University Institutional Review Board. The recording device was an Elekta NeuroMag MEG with 306 channels, consisting of 204 planar gradiometers and 102 magnetometers, set at a 1 ms sampling rate. The data was recorded as the subjects performed a naturalistic reading comprehension task. Specifically, they read chapter 9 of the novel “Harry Potter and the Sorcerer's stone,” which contains 5,176 words. Each word was presented for a fixed duration of 500 ms, and presented using Rapid Serial Visual Presentation (RSVP). The data was preprocessed using a standard precprocessing pipeline, which consists of Maxfilter, SSS, tSSS, EmptyRoom, frequency filtering (high pass 1 Hz, low pass 150 Hz, notch filter of 60 Hz, 120 Hz), heartbeat and blink removal. We downsample the data to 25 ms resolution.

### 2.2. Denoising Framework Using Cross-Subject Predictive Modeling

We formalize the method here—Let there be *N* subjects, denoted as *S*_1_, *S*_2_…*S*_*N*_. For each subject, a typical MEG dataset is a tensor of the form *n*_events_ × *n*_sensors_ × *n*_time_, where *n*_events_, *n*_sensors_ and *n*_time_ correspond to the number of events, sensors, and time points per event. In this formulation, we consider each event to take the same duration and have the same number of time points. For a naturalistic scene recognition task in which pictures of scenes are presented for a fixed duration each, for instance, each picture is an event. In our reading comprehension task, words are shown one at a time for a fixed duration, and each word is an event. If each event is presented with variable duration, then the event data should be padded or truncated to a fixed window after event onset.

We reshape subject *S*_*i*_'s data to a matrix *X*_*i*_ of size *n*_*events*_ × *d*, where *d* is the dimensionality of the *n*_*sensors*_ × *T* flattened MEG vector per event. Either all sensors and all time points per event could be chosen, or some subset of each. Based on the chosen subset of sensors and time points, we obtain different modeling settings described in detail in section 3.1.

We describe 2 approaches to cross-subject modeling: (a) PM, which model the shared response between each pair of subjects in subject space; and (b) SRM, which models a shared response across all subjects in latent space.

#### 2.2.1. Denoising Framework When Using PM

We model a target subject *S*_*t*_'s neural response *X*_*t*_, as a linear function of a source subject *S*_*s*_'s response, *X*_*s*_:


(1) $$\begin{array}{l} X_{t}={W_{t\leftarrow s}}^{⊤}X_{s}+b_{t\leftarrow s}+\epsilon . \end{array}$$

We compute the estimated weights Ŵ_*t*←*s*_ and bias ${\hat{b}}_{t\leftarrow s}$ of this model using multivariate linear Ridge regression and *k*-fold cross validation. Given the noisy nature of single-trial MEG data, we employ ridge regression to avoid high variance in the weights, and consequently overfitting. The regularization parameters for ridge regression are chosen using inner cross-validation within the training folds. Concretely, we split the *n* data points into *k* folds. Since the data is time-series and non-IID in nature, it is important to choose continuous blocks of the data as folds. Shuffled splits can lead to over-optimistic test estimates, due to the temporal correlations present in time series data (Yang et al., 2019). Additionally, the samples at the edges of each fold are temporally adjacent to training data and thus strongly correlated to samples seen in training. To account for this, we designate the data from each run as a fold, and drop 60 samples (30 s) at the beginning and end of the fold. Leaving each such fold *j* out for subject *S*_*i*_ as a test fold, denoted by $X_{i}^{\left(j,test\right)}$, we fit a linear ridge regression model with weight matrix ${Ŵ}_{t\leftarrow s}^{\left(j\right)}$ and bias ${\hat{b}}_{t\leftarrow s}^{\left(j\right)}$ using the rest of the dataset denoted by $X_{i}^{\left(j,train\right)}$.

We use $X_{s}^{\left(j,train\right)}$ and $X_{t}^{\left(j,train\right)}$ to compute the estimated weights ${{Ŵ}_{t\leftarrow s}}^{\left(j\right)}$ and bias ${\hat{b}}_{t\leftarrow s}^{\left(j\right)}$. For target *S*_*t*_ from source subject *S*_*s*_, we compute an estimate of the test fold *j* as


(2) $$\begin{array}{l} {\hat{X}}_{t\leftarrow s}^{\left(j,test\right)}={{Ŵ}_{t\leftarrow s}}^{\left(j\right)⊤}X_{s}^{\left(j,test\right)}+{\hat{b}}_{t\leftarrow s}^{\left(j\right)}. \end{array}$$

We concatenate these test predictions across all test folds *j*, and denote this ${\hat{X}}_{t\leftarrow s}$.


(3) $$\begin{array}{l} {\hat{X}}_{t\leftarrow s}={\text{concat}}_{j}\left[{\hat{X}}_{t\leftarrow s}^{\left(j,test\right)}\right] \end{array}$$

To compute the final denoised neural response of target subject *S*_*t*_, we average its estimates from all other source subjects:


(4) $$\begin{array}{l} {\hat{X}}_{t}=\frac{1}{N-1}\underset{s\neq t}{\sum }{\hat{X}}_{t\leftarrow s} \end{array}$$

**Figure 1:**
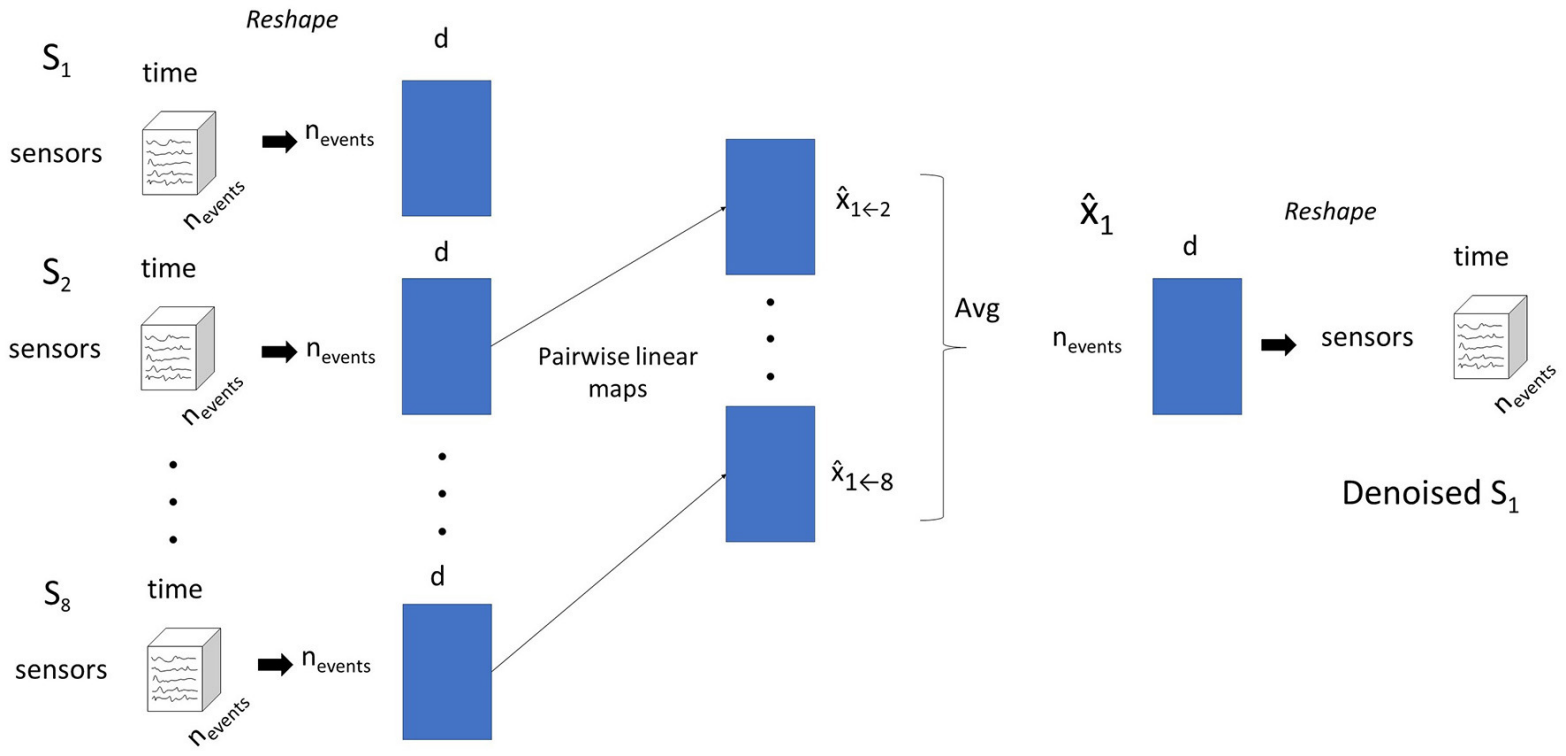
The pairwise mapping (PM) approach for denoising data for subject *S*_1_. Subject data is shaped into a 2-D matrix. Linear models that predict *S*_1_'s data from each other subject's data are estimated. The predictions for *S*_1_'s from all other source subjects are averaged into a single prediction.

The procedure is illustrated in Figure 1.

#### 2.2.2. Denoising Framework When Using SRM

We use the SRM as introduced by Chen et al. (2015) in our framework. The SRM is a factor model, representing each subject's data *X*_*i*_ (size *n*_*events*_ × *d*) as the product of subject-specific basis of topographies *W*_*i*_ (size *L* × *d*), and a *shared* response *Z* (size *n*_*events*_ × *L*) across all subjects that represents the observed stimulus in latent space:


(5) $$\begin{array}{l} \begin{array}{cc} X_{i} & =ZW_{i}+\epsilon_{i}. \end{array} \end{array}$$

Ẑ and Ŵ_*i*_ are chosen to optimize the following objective:


(6) $$\begin{array}{l} \begin{array}{c} {Ŵ}_{i},Ẑ=\underset{{Ŵ}_{i},Ẑ}{\mathrm{argmin}}\underset{i}{\sum }||X_{i}-Ẑ\hat{W_{i}}{||}_{F}^{2}\\ \text{s.t }\hat{W_{i}}{\hat{W_{i}}}^{⊤}=I_{L} \end{array} \end{array}$$

where ||.||_*F*_ denotes the Frobenius norm and *L* is a hyper-parameter corresponding to the dimensionality of the estimated latent space. As in the previous section, for each test fold *j*, we use the rest of the dataset $X_{i}^{\left(j,train\right)}$ in all subjects to estimate the basis of topographies ${Ŵ}_{i}^{\left(j\right)}$ and Ẑ^(*j, train*)^ according to Equation (6). Methods to solve the above optimization problem are described in Chen et al. (2015).

The same basis of topographies ${Ŵ}_{i}^{\left(j\right)}$ are used in both training and test stages. The shared response Ẑ^(*j, train*)^ can be seen as a latent representation of the stimulus in the training data, and is discarded. In the traditional SRM, the shared response of the unseen test data can be estimated from the basis ${Ŵ}_{i}^{\left(j\right)}$ and $X_{i}^{\left(j,test\right)}$ as


(7) $$\begin{array}{l} \begin{array}{c} {Ẑ}^{\left(j,test\right)}=\frac{1}{N}\underset{i}{\sum }X_{i}^{\left(j,test\right)}{{Ŵ}_{i}}^{\left(j\right)⊤}. \end{array} \end{array}$$

However, the principle of our framework involves cross-subject estimation. Thus we modify Equation (7) to produce a latent representation ${Ẑ}_{i}^{\left(j,test\right)}$ for each subject's test fold, that excludes its own test data. We then obtain an estimate of the test data for each subject for that fold, and repeat the process for each fold. The folds are concatenated to produce the final transformed data, as in Equation (8)


(8) $$\begin{array}{l} \begin{array}{c} {Ẑ}_{i}^{\left(j,test\right)}=\frac{1}{N-1}\underset{i\neq k}{\sum }X_{k}^{\left(j,test\right)}{Ŵ}_{k}^{\left(j\right)⊤}\\ {\hat{X}}_{i}^{\left(j\right)}={Ẑ}_{i}^{\left(j,test\right)}{Ŵ}_{i}^{\left(j\right)}\\ {\hat{X}}_{i}={\text{concat}}_{j}\left[{\hat{X}}_{i}^{\left(j\right)}\right] \end{array} \end{array}$$

Conceptually, this differs from the PM model in section 2.2.1 because here *Z* is an averaged response in latent space, while the PM approach considers averaged responses in subject space. There is no theoretical justification for using one over the other, and the method ultimately used in the framework is best chosen through empirical comparison on the dataset under study.

### 2.3. Evaluation in Settings With Low SNR

The *Kv*(2*K*) classification metric is an evaluation metric that is computed given a predictions-matrix, and a gold target matrix, both of size *n*_*samples*_ × *d*. It is a hypothesis-test statistic that has the added benefit of being normalized to range between 0 and 100%. It was proposed to evaluate model predictions in settings with very low SNR, with the null hypothesis being that test predictions are random and have no relation to the targets. First we provide the intuition behind it, using the simplest variant of the *Kv*(2*K*) metric, the 1*v*2:

We randomly pick a predicted sample/row, along with its corresponding gold vector. We also choose a different random predicted sample (which we term the negative sample). Denote these as ${\hat{x}}_{1}$, *x*_1_, and ${\hat{x}}_{2}$ respectively. Then we assign an accuracy of 1.0 for this pair if the gold vector is closer in Euclidean space to the corresponding prediction than the negative sample. Concretely, let *E*(*a, b*) denote the Euclidean distance between vectors *a* and *b*. We assign 1.0 if $E\left(x_{1},{\hat{x}}_{1}\right)<E\left(x_{1},{\hat{x}}_{2}\right)$, and 0.0 if not. We thus compare the distances between 1 gold sample and 2 predicted samples, leading to the term 1*v*2. A large number of such pairs of samples are picked randomly, and average accuracy is reported. This produces an accuracy score that measures how often each gold target vector is closest to its corresponding prediction compared to all other predictions.

To check for statistical significance, we perform permutation testing. In each permutation, we permute the rows of either the prediction matrix or the gold target matrix (so as to break correspondence between them), and then compute the above metric. This gives us an empirical distribution of the scores and helps us detect if the original score we obtained was unusually high, for some chosen *p*-value. Chance accuracy for this metric is typically around 50%. This is intuitive: In the null hypothesis where the predictions are random, each gold vector can be closer to either its corresponding prediction or a random negative sample with equal probability.

The other variants of the *Kv*(2*K*) metric were introduced because of the low SNR, to increase the power of the test. Instead of picking 1 pair of samples as in the 1*v*2 case, we pick *K* pairs. We modify the condition above to use an aggregate of the *K* samples. Illustrated example for 2*v*4, involving 2 gold samples and 4 predicted samples in total:

Pair 1 : ${\hat{x}}_{1}$, *x*_1_; and ${\hat{x}}_{2}$.

Pair 2 : ${\hat{x}}_{3}$, *x*_3_; and ${\hat{x}}_{4}$.

We assign 1.0 if $E\left(x_{1},{\hat{x}}_{1}\right)+E\left(x_{3},{\hat{x}}_{3}\right)<E\left(x_{1},{\hat{x}}_{2}\right)+E\left(x_{3},{\hat{x}}_{4}\right)$, and 0.0 if not. The rest of the procedure is analogous to the 1v2 case.

Finally, the choice of negative samples allows us to control for various factors. As an example, we can choose to use only negative samples corresponding to words having the same length as the positive sample. This eliminates the possibility of using an “easy” factor to discriminate between the samples.

In this work, we use this metric to measure the cross-subject modeling performance of different settings and methods, by comparing the predicted data with the original data. For target *S*_*t*_, source *S*_*s*_, the accuracy *Acc*(*S*_*t*_, *S*_*s*_) of predicting *S*_*t*_ from *S*_*s*_ is predicted across all folds *j* as:


$$Acc\left(S_{t},S_{s}\right)=\frac{1}{k}\overset{k}{\underset{j=1}{\sum }}20v40\left(X_{t\leftarrow s}^{\left(j\right)},X_{t}^{\left(j\right)}\right)$$

and, the average accuracy *Acc*(*S*_*t*_) of predicting *S*_*t*_ from all other sources is:


$$Acc\left(S_{t}\right)=\frac{1}{k}\overset{k}{\underset{j=1}{\sum }}20v40\left({\hat{X}}_{t}^{\left(j\right)},X_{t}^{\left(j\right)}\right).$$

Finally, the *Kv*(2*K*) metric is also used to measure the performance of decoding models trained on the original or denoised data. Decoding models map brain data to stimulus space, and have been used in Brain-Computer Interfaces (Muller-Putz et al., 2016; Willett et al., 2019), as well as a multitude of studies in cognitive neuroscience (Pasley and Knight, 2013; Martin et al., 2014; Holdgraf et al., 2017; Abdou et al., 2021). In this work, we train linear ridge regression models that map the brain data corresponding to a word, to its semantic vector representation obtained from ELMo (Peters et al., 2018), a neural language model, whose representations have been previously shown to correlate significantly with MEG recordings of people reading text (Toneva and Wehbe, 2019; Toneva et al., 2020). The predicted ELMo vectors are compared to the ground-truth ELMo vectors for each word, in the manner described above.

## 3. Results

### 3.1. Cross-Subject Predictive Modeling Can Be Done in Single Trial MEG

First, we investigate the feasibility of cross-subject predictive modeling in single trial MEG data. For a response variable (sensor-time point) in the target subject, we identify different predictors sets in the source subject. One option is to use only the corresponding sensor and time point in the source subject. However, different participants might have some anatomical variability, or have differences in the latencies with which they process information. Therefore, incorporating information from other sensors and timepoints might be beneficial.

**Figure 2:**
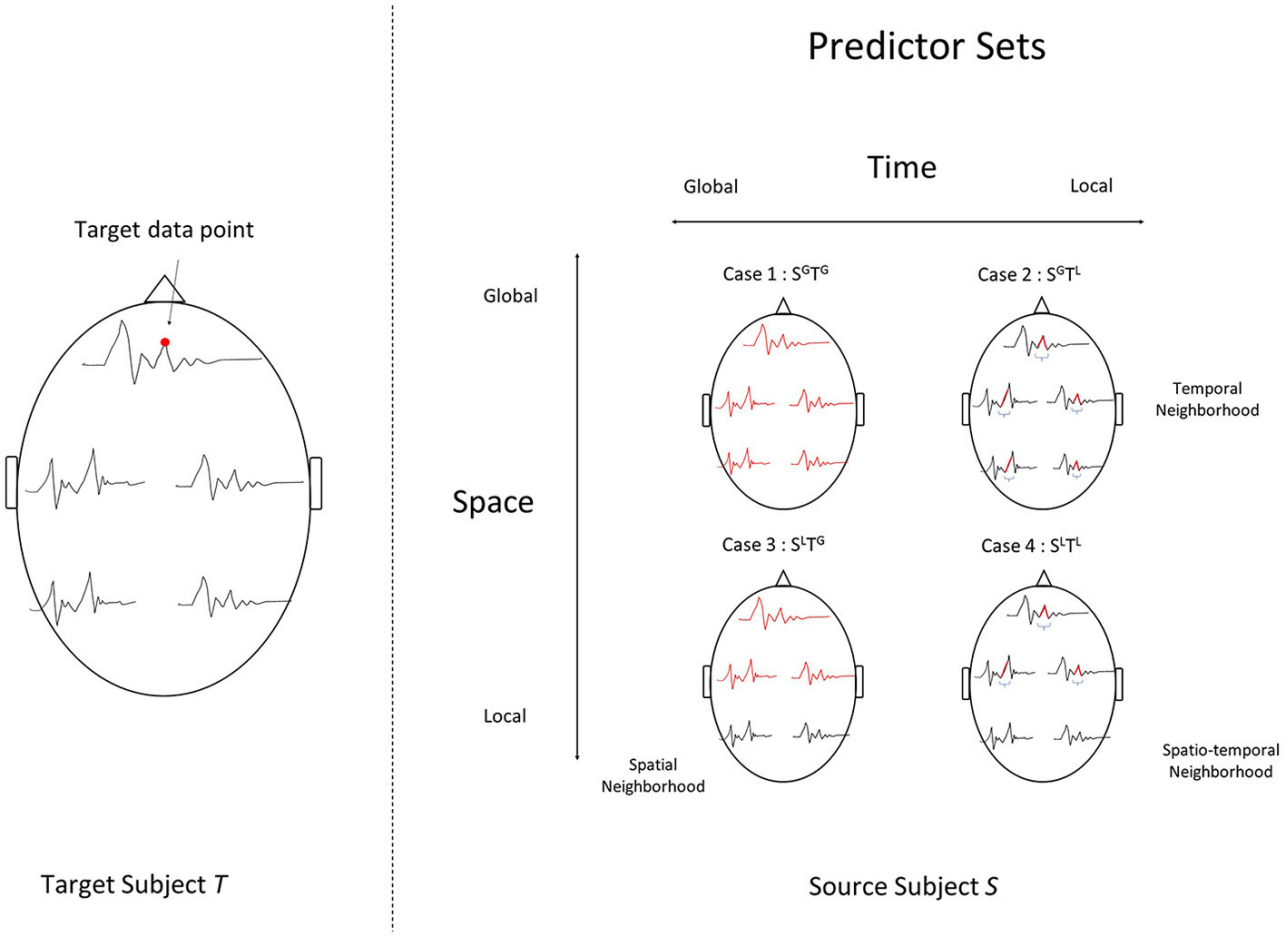
Four modeling settings, where each unit in a target subject's data **(left)** is estimated using different subsets of the source subject's data **(right)**. The chosen set of data points that act as predictors in each setting are highlighted in red on the right. To predict a target data point indicated by the red dot **(left)**, we use : (Case 1) *S*^*G*^*T*^*G*^, a setting with all sensors at all time-points post event-onset. (Case 2) *S*^*G*^*T*^*L*^, a temporal neighborhood around the data point, for all sensors, (Case 3) *S*^*L*^*T*^*G*^, a spatial neighborhood around the data point, and all time points or (Case 4) *S*^*L*^*T*^*L*^, a spatial and temporal neighborhood.

The choice of spatial/temporal neighborhoods that form the set of predictors gives rise to a spectrum of modeling settings that belong to the cross-predictive modeling family. The most *local* type of modeling uses only the corresponding sensor-time point in the source subject. The most *global* type of modeling uses all sensors and all time points after event onset in the source subject as the predictor set. These settings are visually depicted in Figure 2.

For each sensor-time response variable after event onset, we use a linear ridge regression model with the following predictor sets:

**Setting** ***S*^*G*^*T*^*G*^** : All sensors (global) and all time-points (global).

**Setting** ***S*^*G*^*T*^*L*^** : All sensors (global) and a temporal neighborhood (local).

**Setting** ***S*^*L*^*T*^*G*^** : Sensor neighborhood (local) and all time-points (global).

**Setting** ***S*^*L*^*T*^*L*^** : Sensor neighborhood (local) and temporal neighborhood (local).

Each of these settings have different implications when drawing biological inferences, and we discuss this in detail in section 4. For each setting, we compute the 20*v*40 validation accuracy between the predicted data and the original data, averaged across all subjects, shown in Table 1. This serves as a measure of the success of predictive modeling in each setting. We also compare against denoised data from the SRM-based approach, choosing the hyper-parameter *L* through cross-validation. We observe that all settings show higher-than-chance accuracy (0.5), and the deviations from chance are statistically significant (*p* < 0.05). We observe that the PM approach is more accurate than SRM at predicting subject data. We conclude that the PM predictions capture information shared across subjects and could be useful for denoising MEG data.

**Table 1:** 20*v*40 validation accuracies averaged over subjects, in each setting, for Pairwise Mapping (PM) and Shared Response Model (SRM).

**Settings**	**PM Avg 20v40**	**SRM Avg 20v40**
*S* ^*G*^ *T* ^*G*^	0.884 ± 0.021	0.7729 ± 0.017
*S* ^*G*^ *T* ^*L*^	0.8703 ± 0.013	0.7735 ± 0.012
*S* ^*L*^ *T* ^*G*^	0.8767 ± 0.019	0.7628 ± 0.018
*S* ^*L*^ *T* ^*L*^	0.859 ± 0.023	0.7771 ± 0.014

### 3.2. Estimates From Source Subjects Should Be Averaged

In our denoising framework, for each target subject, we obtain multiple estimates, one from each source subject. We investigate how the modeling performance varied for each target-source pair, and how best to combine the estimates of a target from different source subjects.

**Figure 3:**
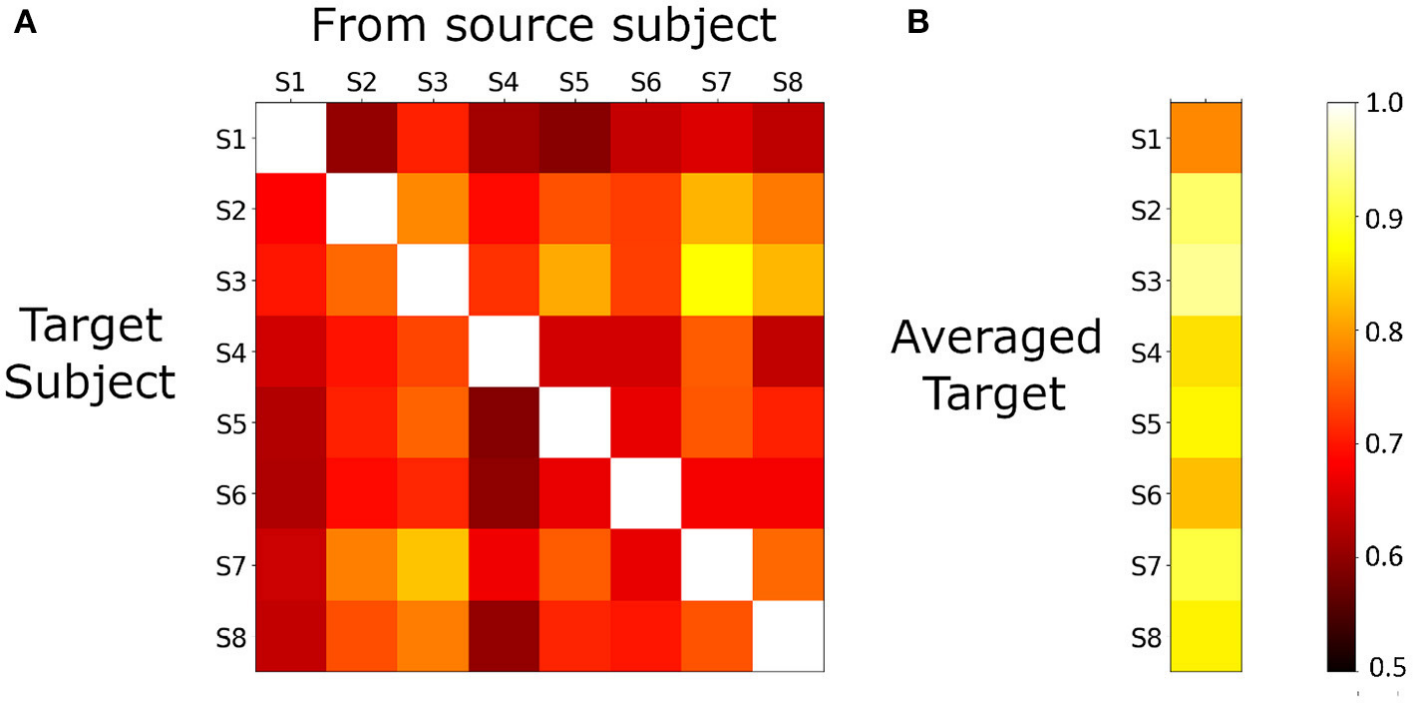
**(A)**, 20*v*40 validation accuracy of PM predictions for each source-target model (using the *S*^*G*^*T*^*G*^ setting). All accuracies are greater than chance (0.5), and statistically significant (*p* < 0.05), indicating that cross-subject modeling in single-trial MEG is feasible. **(B)**, for each target subject, estimates from all sources are averaged and 20*v*40 validation accuracy is computed. Note that each accuracy is greater than any of the individual estimates from any source, indicating that averaging improves the quality of the denoised data.

Figure 3A shows the matrix of 20*v*40 validation accuracies for each (target, source) subject pair. Each row represents a target subject when estimated from all other subjects. Chance accuracy is 50%, and all predictions are above chance accuracy and statistically significant (*p* < 0.05). A natural way to combine the different estimates of a single target is to average them. We observe from Figure 3B that the averaged estimates for each target shows higher validation accuracies than any of the individual estimates for that target.

We also consider the modeling performance across target subjects, and its implications on the quality of each subject's data. We observe that subject *S*_3_ and subject *S*_8_ show uniformly higher values than other subjects, and the highest accuracy appears when *S*_3_ is the target and *S*_8_ is the source. In addition to tests for measuring task-compliance, this framework can serve as an important tool in measuring the quality of data across subjects.

### 3.3. The Cross-Subject Modeling Is a Denoising Transformation

We hypothesize that the PM transformation retains the task-related signal in the MEG data, while reducing noise. In this section, we perform experiments to confirm this denoising hypothesis, by demonstrating that the transformation preserves (and enhances) task-specific signal. We demonstrate this in four steps. First, we present a visual analysis of the original data, contrasted with the PM/SRM transformed data, which shows a more systematic and clear pattern. Second, we compare the semantic decoding performance of the original and transformed data. Third, we compare the ability to model the N400 response in the data. Fourth, we present results from encoding models of the original and transformed data.

#### 3.3.1. PM Transformed Data Shows a Regular Pattern

**Figure 4:**
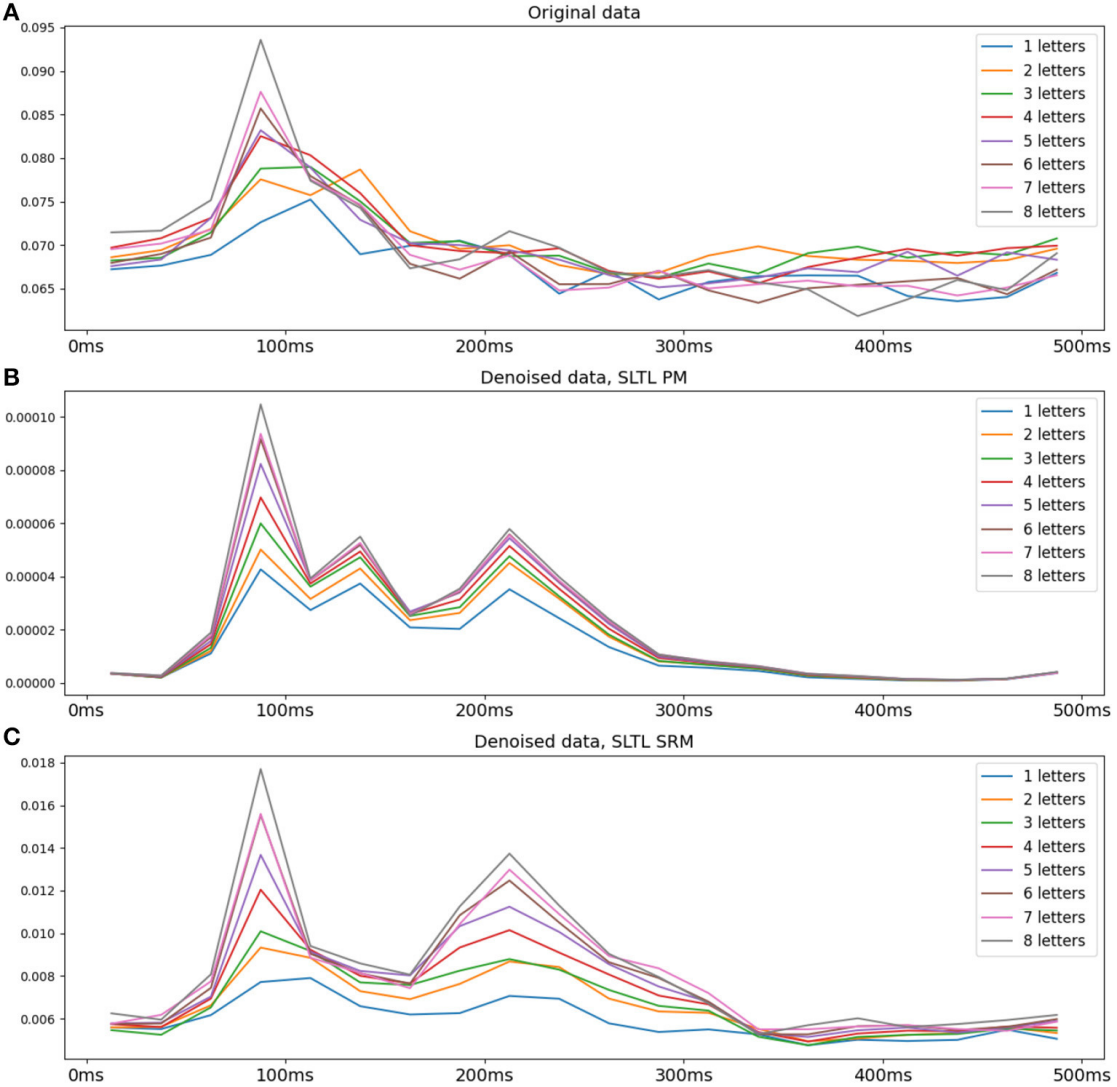
Average signal power for words with different numbers of letters for the original data and the PM and SRM denoised data. The data is squared and averaged over gradiometer sensors in the occipital region, over words of each length, and over all subjects. **(A)** Original data, **(B)** Setting *S*^*L*^*T*^*L*^ PM denoised, **(C)** Setting *S*^*L*^*T*^*L*^ SRM denoised. The PM denoised data shows a regular gradation in power proportionate to the number of letters. Both the original data and the SRM denoised data also suggest such a pattern, but is most visible in the PM denoised data.

We observe a regular pattern when we split the dataset into groups of words of the same length, and average within these groups. Specifically, the data is squared and the time courses following word-onset are averaged across sensors to obtain the power of the signal. The result is averaged across words of each group. Figure 4 shows this post-word-onset time series for the original and *S*^*L*^*T*^*L*^ setting denoised data, averaged over subjects, using both PM as well as the SRM. Each line represents the average power for words of a certain length, across all sensors. A clear vertical gradation is observed in the transformed data, indicating a direct proportional relationship between word length and the power of the MEG signal (and consequently the underlying neural activity). We observe increasing hints of this pattern in the raw data and the SRM-denoised data, but this is much clearer in the PM-denoised data. Overall, a visual inspection of the transformed data suggests a certain regularity and structure.

#### 3.3.2. Transformed Data Shows Higher Semantic Decoding Performance

**Figure 5:**
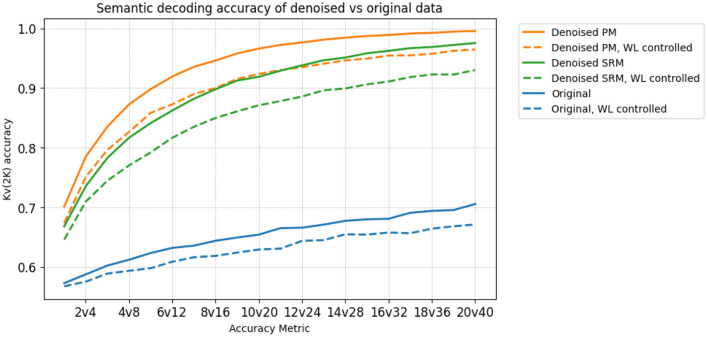
The *Kv*(2*K*) accuracies of decoding models trained on the denoised PM data (orange), the denoised SRM data (green) and the original data (blue), before/after controlling for Word Length (WL). The *S*^*G*^*T*^*G*^ setting is used for both PM and SRM. In both cases, PM is uniformly higher than SRM, which in turn is uniformly higher than Original. The denoised data also begins saturating at earlier variants of *Kv*(2*K*), indicating a stronger SNR. Furthermore, controlling for word length reduces decoding accuracy in all 3 datasets as expected, but still demonstrates significantly high (*p* < 0.05) accuracies, indicating semantic information beyond WL is preserved in the denoised data.

We investigate if the transformed data preserves the semantic information present in the original MEG data. To that end, we use word-level embeddings from ELMo (Peters et al., 2018), a pre-trained language model, to model the individual word meaning. We extract the first level, non-contextualized ELMo embeddings that correspond to each word. We then train two ridge regression models for decoding. The hyperparameters for ridge regression are chosen using cross-validation within each training fold. One maps the original MEG data to token level ELMo embeddings, and the other maps the transformed data to the token-level ELMo embeddings of the words corresponding to the MEG data. We then measure the performance of each decoding model using variants of the 20*v*40 metric, such as 1*v*2, 2*v*4, … up to 20*v*40. Each higher variant is used to detect weaker SNRs. Thus, for a dataset that exhibits high SNR, we expect the decoding accuracy to begin saturating at earlier variants of *Kv*(2*K*). We also control for number of letters, to investigate if the high decoding accuracy is obtained purely by correctly predicting word length information, or some confound associated to it. For example, saccadic activity has been shown to act as a confound (Muthukumaraswamy, 2013), being highly correlated with word length. We control for word length by modifying the *Kv*(2*K*) accuracy metrics to sample negatives only from words of the same length. Being able to differentiate between the brain data of these pairs of words indicates that there is some information beyond word length embedded in it. The decoding accuracies averaged across subjects is shown in Figure 5. We observe that the PM method leads to better decoding accuracy than using the original data and the SRM method, across all choices of *K*.

#### 3.3.3. Transformed Data Shows Stronger Correlation Between N400m Effect and Word Surprisal

The N400 is a human event related potential (ERP) that arises in response to semantic incongruity in a wide range of stimulus types: written and spoken words, drawings, photos, and videos of faces, objects and actions, and mathematical symbols (Kutas and Federmeier, 2011). It is characterized by a negative deflection that occurs sometime during the 300–500 ms period after word onset. The N400 can also been observed in MEG, referred to as the N400m (Parviz et al., 2011). In reading comprehension tasks, one measure of semantic congruity is word surprisal, expressed as the negative log of the (one-sided) cloze probability of word *w*_*t*_ with respect to the previous words:


$${\text{Surprisal}}_{w_{t}}=-\log p\left(w_{t}|w_{t-1},w_{t-2},\ldots w_{0}\right).$$

It has been shown that the N400 response is graded by the magnitude of semantic surprisal. Word surprisal estimates using language models was found to correlate with the magnitude of the N400 (Frank et al., 2013) and N400m response (Parviz et al., 2011). We use a Recurrent Neural Network model trained on a large Harry Potter fan-fiction corpus by Wehbe et al. (2014b) to estimate the word surprisal for every word in our dataset. For the 5,176 words in the original and transformed datasets, we also estimate the N400m response. We employ the *S*^*L*^*T*^*L*^ setting because it allows inference in both space and time, which is required to identify the brain region and magnitude of the N400m response accurately (discussed in section 4). For each sensor in a localized brain region, the MEG data points from the 300–500 ms time period are averaged, and the N400m response is estimated by max pooling across the sensors in that brain region.

As shown in Table 2, we find that the temporal and left-parietal regions show statistically significant (*p* < 0.05) correlation between the N400m response and word surprisal in both the original and denoised data, but significantly higher in the PM denoised MEG data. This suggests that the denoised data allows more accurate decoding of effects such as semantic surprisal.

**Table 2:** Correlation of N400m magnitude with word surprisal estimated by an RNN, across various brain regions.

**Brain region**	**Correlation original**	**Correlation PM transformed**
left-temporal	0.089 (*p* = 1e-4)	**0.266** (*p* = 1e-4)
right-temporal	0.038 (*p* = 0.0014)	**0.12** (*p* = 1e-4)
left-parietal	0.027 (*p* = 0.0132)	**0.107** (*p* = 1e-4)
right-parietal	0.0014 (*p* = 0.33)	0.034 (*p* = 1e-4)
left-occipital	−0.014 (*p* = 0.76)	0.044 (*p* = 1e-4)
right-occipital	−0.016 (*p* = 0.82)	0.074 (*p* = 1e-4)

*The bold values indicate brain regions of our transformed data outperforms the original, among the rows where they are both significant (p < 0.05)*.

#### 3.3.4. Transformed Data Shows Better Encoding Model Performance

**Figure 6:**
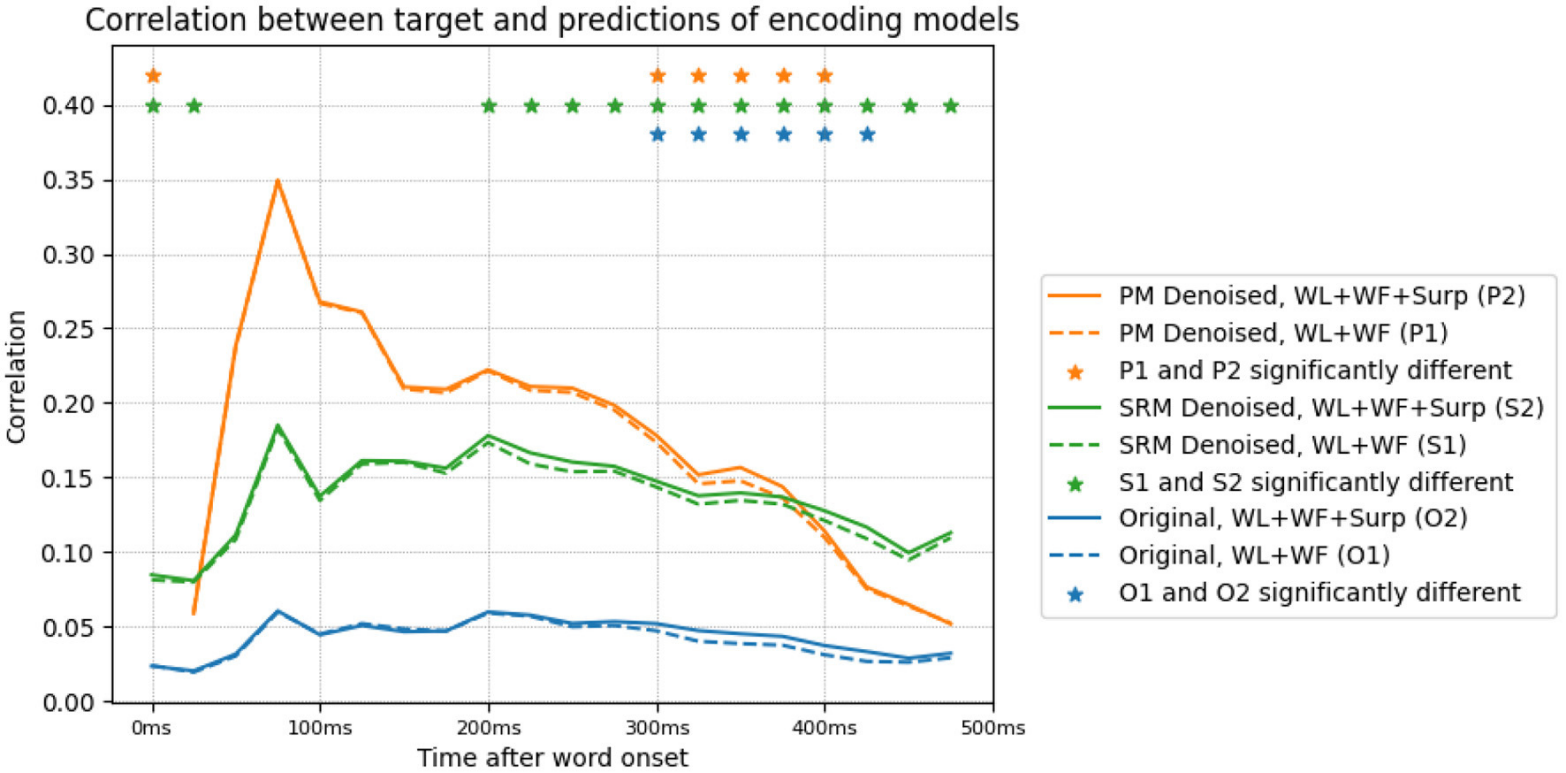
Prediction performance (measured through Pearson correlation) of various encoding models trained on the original and denoised data. The encoding models are trained using word length (WL), frequency (WF) and surprisal (Surp) features. The correlation of encoding model predictions with the target data, for original and denoised data, with/without surprisal is shown. Denoising is performed with the *S*^*L*^*T*^*L*^ setting, and results in significantly higher encoding performance. The inclusion of surprisal improves the correlation between encoding model predictions and target data in both denoised and original settings, in the time bins corresponding to N400m (300–500 ms).

We lastly study the effect of transforming the data on encoding model performance. We train an encoding model of the original and denoised data using word length, log-transformed word frequency and surprisal as predictors. We present the results in Figure 6. The three features predict the MEG activity starting soon after word onset and lasting until the end of word presentation. We observe that the PM transformed data has a prediction performance that is significantly higher than the original data. This is likely due to the fact that the data that is predicted has a higher SNR, and consequently the ability to predict the signal is greatly improved. This is also the case with SRM denoised data.

Surprisal is highly anti-correlated with word length and word frequency. We perform experiments to investigate if the N400m effect's correlation with surprisal can be explained away by these word length and frequency features. We train another pair of encoding models, after excluding the surprisal features. Figure 6 shows the decrease in the correlation of the encoding model's predictions for each time point, after excluding these surprisal features. This difference corresponds to the amount of activity that is uniquely predicted by surprisal, and appears to be peak (with significance, *p* < 0.05) around 300–400 ms after word onset in both the original and transformed data.

## 4. Discussion

In this paper, we introduced a framework for denoising MEG data by relying on multiple subjects' data instead of multiple repetitions of the stimulus, as well as Pairwise Mapping (PM), a cross-subject modeling technique that can be used in the framework. We also adapted an existing method (SRM) to fit into the above framework. Denoising brain data using this framework also allowed an easier interpretation of patterns in the signal, and higher decoding and encoding accuracy. The framework is therefore a useful tool in the arsenal of computational neuroscientists who work with MEG data. In our dataset, we found that PM predicted held-out data for one participant from others with higher accuracy than SRM. The denoised data obtained using PM also exhibited higher encoding and decoding performance than SRM. However, we should note that since PM and SRM share conceptual similarities, this trend may not hold over all datasets. While PM is a simpler procedure that does not require choosing a latent embedding size, it is unclear if this is responsible for the observed trend in our dataset. Ultimately, the choice of the modeling method used in our framework should be made based on empirical results on the dataset under study. Next, we discuss the significance and limitations of our findings.

One potential confound in MEG data is eye movement (Muthukumaraswamy, 2013) and our experiments were designed to account for this. First, the words were presented centered on the screen, minimizing eye movements. Second, we performed preprocessing to remove eye movement artifacts (mainly blinks). Most importantly, however, we controlled for word length (WL) in our experiments. As mentioned previously, we accounted for both word length (WL) and any confounding factors correlated with it (such as saccadic movements). In the decoding experiment results, we selected negative samples corresponding to words of exactly the same length as the positive sample, and required that they be separable using the brain data. To compute the *Kv*(2*K*) score, each pair of ${\hat{x}}_{1}$ and ${\hat{x}}_{2}$ were chosen such that they corresponded to 2 words of the same length, e.g., “Harry” and “There.” Figure 5 (and caption) shows that this differentiation can be done with significantly high accuracy (dashed-line plots, *p* < 0.05). We expect saccadic activity among a set of words of the same length to be similar (for “Harry” and “There” in the running example). Being able to differentiate between their data indicates the presence of some information beyond word length as well as possible confounds correlated with this equal word length.

Regarding the choice of modeling settings, it is possible that settings with richer predictor sets (e.g., *S*^*G*^*T*^*G*^ compared to *S*^*L*^*T*^*L*^) lead to more successful prediction/modeling. However, there are compelling reasons not to simply choose the setting that provides the best modeling performance. The end goal is not to predict the data, but rather to study the brain and draw conclusions relevant to cognitive neuroscience, many of which are related to specific locations and timings. Consider for instance that we want to make a statement about a specific processing latency in the brain, then we cannot use the *S*^*G*^*T*^*G*^ or the *S*^*L*^*T*^*G*^ setting because then each time point in the denoised data will contain information from other time points (in other subjects). We would not be able to make inferences about timing using this setting. Similarly, if we are interested in spatial inference, we should not use the *S*^*G*^*T*^*G*^ or the *S*^*G*^*T*^*L*^ settings, as they could lead to information leak between different brain regions.

We suggest that the denoising setting be selected based on the study of interest, to achieve maximum modeling ability without interfering with the type of inference to be made. Table 3 shows the different types of inference that are allowed under each setting.

**Table 3:** Types of inference for each setting.

**Settings**	**Type of inference allowed**
Setting *S*^*G*^*T*^*G*^	Global inference
Setting *S*^*G*^*T*^*L*^	Temporal inference
Setting *S*^*L*^*T*^*G*^	Spatial inference
Setting *S*^*L*^*T*^*L*^	Spatio-temporal inference

The use of our framework imposes some limitations on the conclusions that can be drawn from a study. If an effect is not present in the denoised data, that may be either because it was absent in the original data, or the denoising transformation did not retain it from the original data. No conclusion can be drawn in this case, i.e, the absence of the effect in our denoised data does not prove that the effect was absent in the underlying brain data. Since we have seen that the denoising procedure has improved and not reduced our ability to model reading processes, we conjecture that studying other cognitive processes would be made easier after denoising with the framework.

While MEG is the modality that is most benefited by such a method (due to low SNR), the denoising procedure can be applied to other imaging modalities, provided multiple subjects are exposed to the same stimuli. A related cross-subject prediction approach has been employed with ECoG and fMRI data to estimate a noise ceiling (Schrimpf et al., 2020). Conceptually, these approaches differ in that Schrimpf et al. (2020) computes one noise ceiling estimate per dataset which is then used to calibrate different encoding model performances, while our cross-subject modeling framework is designed to produce a denoised estimate of each subject in the dataset. The utility of our denoising framework on fMRI data will be explored in future work.

## Data Availability Statement

The raw data supporting the conclusions of this article will be made available by the authors, without undue reservation.

## Ethics Statement

The studies involving human participants were reviewed and approved by Carnegie Mellon University and University of Pittsburgh Institutional Review Boards. The patients/participants provided their written informed consent to participate in this study.

## Author Contributions

The data was acquired in previous work by LW. SR developed the framework to denoise data and conducted subsequent analyses. All authors helped conceive and design the computational analyses, contributed to the original draft of the manuscript, as well as review and editing.

## Funding

This research was supported in part by start-up funds in the Machine Learning Department at Carnegie Mellon University, and the Google 2018 Faculty Research Award.

## Conflict of Interest

SR was employed by the company IBM-Research. The remaining authors declare that the research was conducted in the absence of any commercial or financial relationships that could be construed as a potential conflict of interest.

## Publisher's Note

All claims expressed in this article are solely those of the authors and do not necessarily represent those of their affiliated organizations, or those of the publisher, the editors and the reviewers. Any product that may be evaluated in this article, or claim that may be made by its manufacturer, is not guaranteed or endorsed by the publisher.
