# Supplementary material for: Single-Trial MEG Data Can Be Denoised Through Cross-Subject Predictive Modeling
Source: Front Comput Neurosci. 2021 Nov 11;15:737324. doi: 10.3389/fncom.2021.737324 (PMC8632362; doi:10.3389/fncom.2021.737324)
Supplement: Supplementary file 1 [file Data_Sheet_1.pdf]

Single-Trial MEG Data Can Be Denoised Through Cross-Subject Predictive Modeling

Srinivas Ravishankar[1], **Mariya Toneva**[2,3] **and Leila Wehbe**[2,3]
[1]**IBM-Research, NY, USA**
[2]**Machine Learning Department, Carnegie Mellon University, Pittsburgh, PA, USA**
[3]**Neuroscience Institute, Carnegie Mellon University, Pittsburgh, PA, USA**

1 APPENDIX

1.1 Comparison with Unsupervised Transfer Learning approaches

Among TL approaches, the one closest in spirit to our goal is the Euclidean-space Alignment (EA) approach. We perform the EA procedure on our dataset and show the (a) visual analysis to inspect gradation pattern (b) comparison of decoding performance from the EA-aligned data; in the same manner as the decoding analyses described previously.

1.1.1 Visual Analysis

We investigate if the EA-aligned data exhibits the same systematic gradation in power as the word length increases. The comparison with the denoised and original data is shown in Figure A1. The gradation as seen in the denoising framework output does not appear in the EA-aligned data.

1.1.2 Decoding performance comparison

The decoding experimental procedure is identical to the one described previously, performed on the EA-aligned data to investigate its decoding performance. The results of the comparison against the denoised data are shown in Figure A2. Notably, despite the unsupervised nature of the procedure, the EA-aligned data does exhibit higher decoding performance than the original data. However, it does not appear to perform as well as supervised procedures; which agrees with our expectation.

1.2 Visual analysis of N400m time-series trace

Here we attempt to visually contrast the N400m time-trace in congruous and incongruous conditions. Since our experimental design involves a continuous naturalistic reading task, we do not explicitly have contrasting conditions in our setting. We have attempted to approximate it using the surprisal from a neural language model. Concretely, we choose words having > 95 percentile surprisal as incongruous words, and < 5 percentile as congruous words. The data corresponding to these words are averaged, and max pooling is done over sensors. Figure A3 shows the zoomed-in 300-500 ms time range most relevant to the N400m trace, and Figure A4 shows the entire 0-500 ms time range. The N400m effect for incongruous words is visible in both the original as well as the denoised data. It is arguably more systematic in the denoised data, but we leave this to the qualitative judgement of the reader.

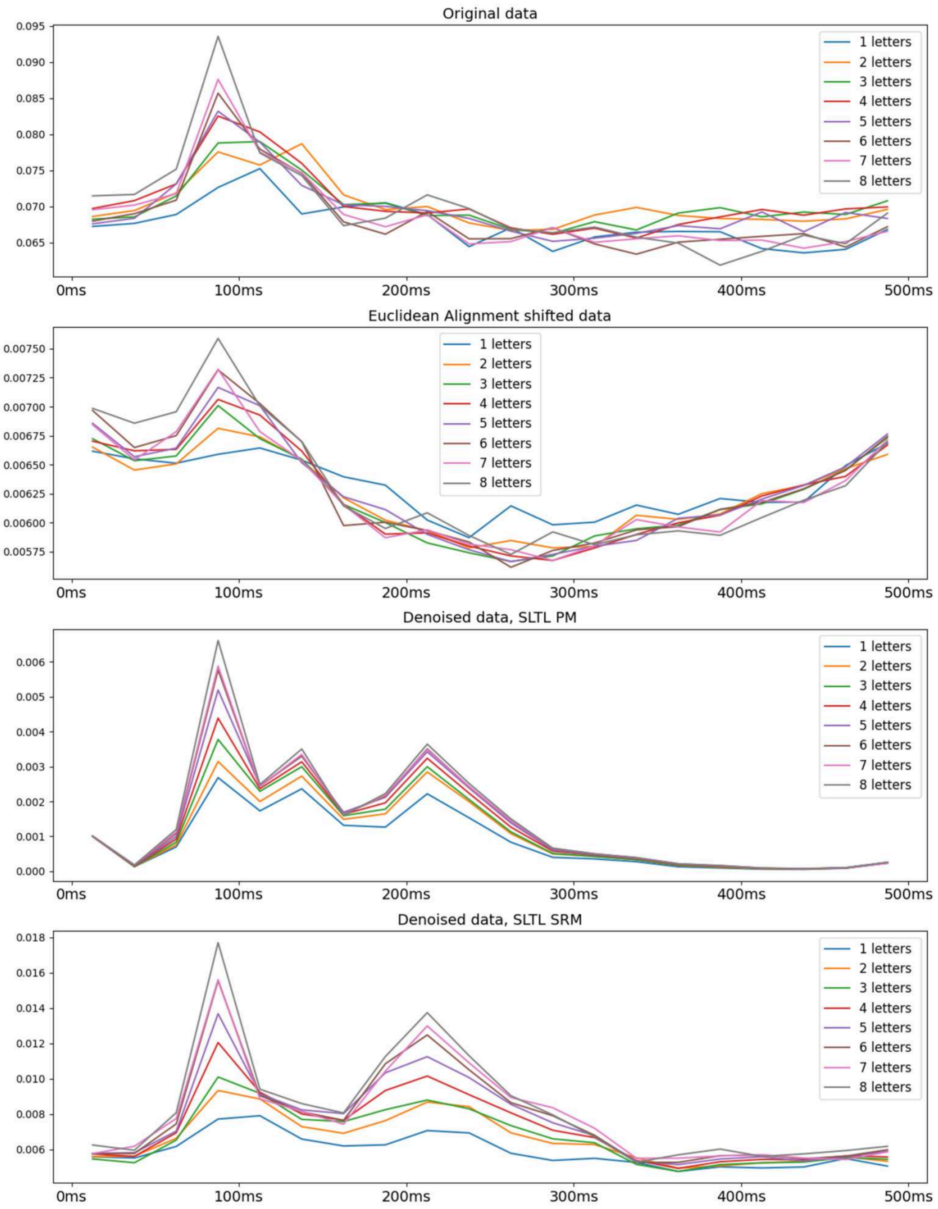

Figure A1. Average signal power for words with different numbers of letters for the original data and the PM and SRM denoised data. The data is squared and averaged over gradiometer sensors in the occipital region, over words of each length, and over all subjects. (a) Original data (b) EA-shifted data (c) Setting $S^L T^L$ PM denoised (d) Setting $S^L T^L$ SRM denoised. The PM denoised data shows a regular gradation in power proportionate to the number of letters. Both the original data and the SRM denoised data also suggest such a pattern, but is most visible in the PM denoised data.

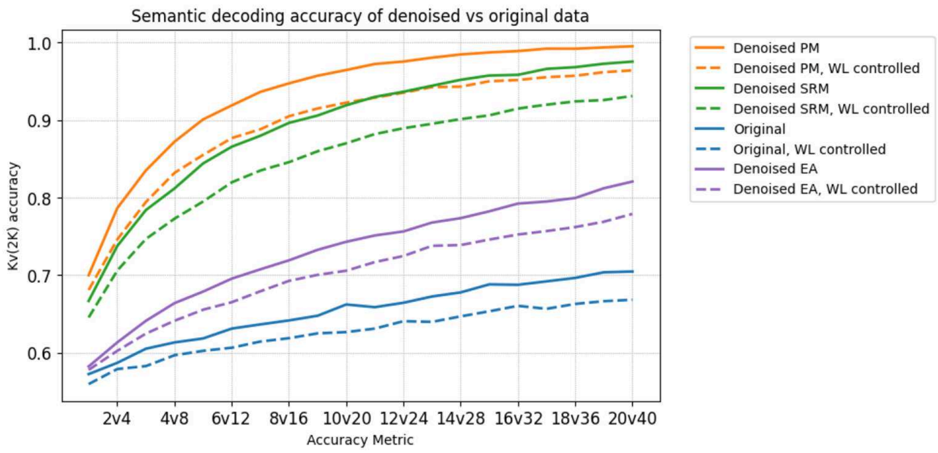

Figure A2. The $Kv(2K)$ accuracies of decoding models trained on the denoised PM data (orange), the denoised SRM data (green), denoised EA data (purple), and the original data (blue), before/after controlling for Word Length (WL). In both cases, PM is uniformly higher than SRM, which in turn is uniformly higher than EA, which is yet again uniformly higher than Original. The denoised data also begins saturating at earlier variants of $Kv(2K)$, indicating a stronger SNR. Furthermore, controlling for word length reduces decoding accuracy in all 4 datasets as expected, but still demonstrates significantly high $(p < 0.05)$ accuracies, indicating semantic information beyond WL is preserved in the denoised data.

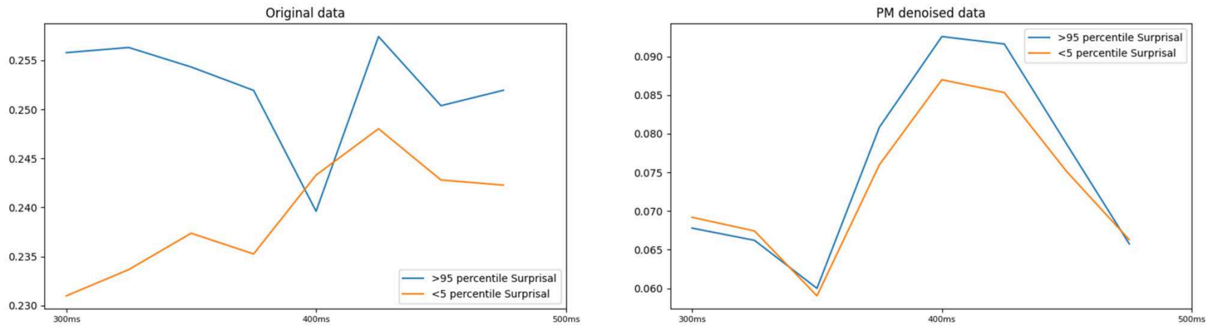

Figure A3. Approximation of contrasting conditions to inspect N400m: > 95 percentile surprisal are considered incongruent words, and < 5 percentile are considered congruent words. Time series is averaged over the words in each condition, and max pooled over sensors.

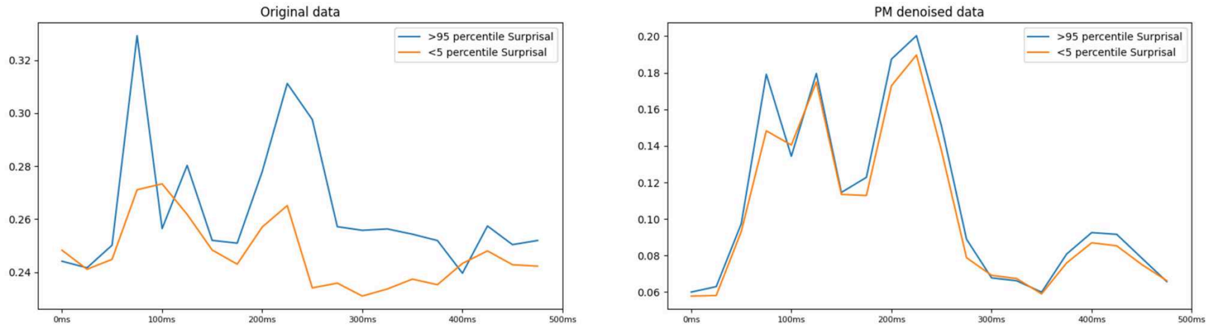

Figure A4. Approximation of contrasting conditions to inspect N400m: > 95 percentile surprisal are considered incongruent words, and < 5 percentile are considered congruent words. Time series is averaged over the words in each condition, and max pooled over sensors; displayed for 500ms post word onset.
